# The EnzymeTracker: an open-source laboratory information management system for sample tracking

**DOI:** 10.1186/1471-2105-13-15

**Published:** 2012-01-26

**Authors:** Thomas Triplet, Gregory Butler

**Affiliations:** 1 Department of Computer Science and Software Engineering, Concordia University, 1455 De Maisonneuve Blvd. West, Montreal, Quebec, H3G 1M8, Canada; 2 Centre for Structural and Functional Genomics, 7141 Sherbrooke Street West, Montreal, Quebec, H4B 1R6, Canada

## Abstract

**Background:**

In many laboratories, researchers store experimental data on their own workstation using spreadsheets. However, this approach poses a number of problems, ranging from sharing issues to inefficient data-mining. Standard spreadsheets are also error-prone, as data do not undergo any validation process. To overcome spreadsheets inherent limitations, a number of proprietary systems have been developed, which laboratories need to pay expensive license fees for. Those costs are usually prohibitive for most laboratories and prevent scientists from benefiting from more sophisticated data management systems.

**Results:**

In this paper, we propose the EnzymeTracker, a web-based laboratory information management system for sample tracking, as an open-source and flexible alternative that aims at facilitating entry, mining and sharing of experimental biological data. The EnzymeTracker features online spreadsheets and tools for monitoring numerous experiments conducted by several collaborators to identify and characterize samples. It also provides libraries of shared data such as protocols, and administration tools for data access control using OpenID and user/team management. Our system relies on a database management system for efficient data indexing and management and a user-friendly AJAX interface that can be accessed over the Internet. The EnzymeTracker facilitates data entry by dynamically suggesting entries and providing smart data-mining tools to effectively retrieve data. Our system features a number of tools to visualize and annotate experimental data, and export highly customizable reports. It also supports QR matrix barcoding to facilitate sample tracking.

**Conclusions:**

The EnzymeTracker was designed to be easy to use and offers many benefits over spreadsheets, thus presenting the characteristics required to facilitate acceptance by the scientific community. It has been successfully used for 20 months on a daily basis by over 50 scientists. The EnzymeTracker is freely available online at http://cubique.fungalgenomics.ca/enzymedb/index.html under the GNU GPLv3 license.

## Background

Spreadsheets are broadly used by the scientific community. Their intuitive and easily understandable user interface is a significant advantage. They are also visually appealing and feature a number of tools to visualize data using charts. Hence, spreadsheets are currently the primary means to store both experimental and manually curated genomic/proteomic data in most laboratories.

### Spreadsheet/Database Paradigm

#### Scalability

Spreadsheets might be sufficient when one needs to organize simple data. However, this approach raises a number of problems as spreadsheets present numerous well-known deficiencies compared to databases when dealing with involved data. As reported in previous studies [1-4], spreadsheets do not scale up well and, as the spreadsheet will expand to accommodate a growing number of records of increasing complexity, data handling -- from data entry to data mining and analysis -- will become increasingly cumbersome, hence reducing the utility of potentially valuable information.

Spreadsheets are also inefficient to handle sparse data, both in terms of storage and performance. Storage is less of a concern nowadays as costs have dramatically decreased in the past few years. However, it should still be taken into consideration when handling millions of records, as is often the case in bioinformatics and large-scale studies in general. In contrast, optimized databases lead to speed improvements.

#### Quality Control

Besides the scalability issue, spreadsheets are subject to data redundancy and consequently data integrity loss. For example, if protein annotations should be displayed in different spreadsheets, they will most likely be duplicated in each document. When an annotation is updated in one place, all occurrences elsewhere may not be updated, which will result in multiple inconsistent versions of the same data. In some cases, determining which versions are obsolete and which version is correct is challenging as different sections of the spreadsheet may have been updated at different times, which can lead to data consolidation issues. Moreover, unlike databases, spreadsheets do not enforce referential integrity: they do not check that resources referenced somewhere in the spreadsheet are still valid, which may be critical, in particular when those resources are frequently updated or deleted.

Spreadsheets are also error-prone and do not facilitate data entry. Typically, any cell can contain any type of data and validation is optional at best. Spreadsheets may even incorrectly infer a data type based on the data, in particular numbers and dates in Excel. For example, the pH range 5 - 6 is interpreted by Excel as the date *May 6th*, and automatically modified and displayed as such when the user types the value in a cell, without even notifying the user.

### Data Sharing and Access Control

Over the past few years, the scientific community has shifted its focus from research project conducted by a single investigator to collaborations among teams of investigators. Projects have effectively become increasingly complex and require more resources to be successful [5,6]. While collaborative research is not new, it raises a number of sharing issues that need to be addressed to maximize the impact of collaborations. One of the keys to successful collaborative research is the development of a centralized system that manages all project data and serves as a unique entry point for the integration of data for data exploration and analysis for all collaborators [7].

However, sharing data using spreadsheets as is commonly done in small groups, proved to be difficult, when possible. For example, a shared Excel spreadsheet can be checked-out and edited by only one user at a time. Other collaborators can only display a read-only copy of the document until changes are committed by the first user. Neither waiting for a user to complete his work or duplicating resources is a practical satisfactory solution.

Finally, spreadsheets provide little -- if any -- security or access control mechanisms. Spreadsheets can be password-protected. However, the password of the spreadsheet is unique and known by many users, and they do not offer the possibility to select what users or groups of users can see/edit in the document: once opened, any record can be displayed by the user. The password is also embedded within the document and it is therefore not possible to revoke access remotely. Databases on the other hand provide advanced access control mechanisms, and enable system administrators to precisely grant or revoke permissions to users or groups of users to create, view, update or delete resources as needed.

### Technology Acceptance Issue

Despite their deficiencies, spreadsheets have been heavily used by biologists because they offer an intuitive and generic user interface that is applicable to most of their projects. Upgrading from spreadsheets to a more sophisticated LIMS is not trivial. To be broadly accepted by the scientific community as a valuable replacement for spreadsheets, LIMS need to present the five acceptance characteristics defined by Rogers [8]:

• *relative advantage*: the extent to which the LIMS offers improvements over spreadsheets,

• *compatibility*: its consistency with social practices and norms among its users,

• *complexity*: its ease of use or learning,

• *trialability*: the opportunity to try an innovation before committing to use it,

• *observability*: the extent to which the technology's gains are clear to see.

### Related Work

To overcome spreadsheets limited capabilities, a number of proprietary LIMS have been developed, for example LABVANTAGE^® ^SAPPHIRE [9], Exemplar LIMS [10] or STARLIMS [11]. However, their licenses fees are usually prohibitive, even for a single user. Moreover, extra features - or modules - usually come at additional costs. As an example, LABVANTAGE is charging a 87,954.75USD^*a *^fee to enable the "Advanced Storage and Logistics" features. STARLIMS is charging an extra 9,571.25USD^*a *^per user for the basic edition of the document management module, 9,571.25USD^*a *^per user for batch processing and 33,499.38USD^*a *^for their Web services framework. Those license fees were obtained on GSA on September 19, 2011 and represent the lowest possible prices negotiated with the U.S. government and may not even be available to non-governmental companies or organizations.

Such costs are clearly prohibitive for most laboratories and therefore restrict the intended audience of these tools to bigger laboratories or to the industry. While numerous open-source LIMS have been developed [12], a large number of existing systems focus on stock inventory and order management and were not designed to track experimental biological results. In addition, many projects were still in very early development stages, not supported any more, or not stable enough to run properly (see Additional File 1 for details). As a consequence, very few systems [13,14] are *effectively *available to the general scientific community. The main goal of the EnzymeTracker is to provide a free and simple open-source alternative.

#### Cloud-based Services

Cloud-based solutions, such as Google Docs [15], Microsoft Office Web Apps [16] or Zoho Office Suite [17], have recently emerged and partially address the limitations of traditional spreadsheets. Those solutions offer a group the possibility to create, edit and save spreadsheets online. They improve access control and versioning mechanisms. Another benefit over traditional spreadsheets is that a unique copy of the spreadsheet is created, which can be accessed and edited online at the same time by many collaborators, thereby solving some of the data consolidation issues.

However, they suffer from the same limitations as traditional spreadsheets in terms of scalability and data quality control as they offer no means to validate data upon entry and no specific features for biological projects. Storage capacity is limited to a few gigabytes at most, which make those solutions inappropriate for large projects. Their graphical web-based user interfaces are dynamic, they are also less sophisticated that typical desktop applications and raised numerous complaints from our collaborators when first introduced. In addition, it should be noted that data are stored on the servers of the service provider, which can raise a number of issues if data confidentially is critical as is often the case in the biomedical field. The reliability of infrastructures supporting could services has been questioned lately, as all of the major service providers have recently experienced at least some glitches, from service outage [18] to data losses [19].

#### iLAP

Stocker et al. [13] recently developed iLAP, a workflow-driven software for experimental protocol development, data acquisition and analysis. iLAP relies on a relational database and a web-based interface to effectively manage complex workflows derived from biological experimental protocols. Integration of external programs using Java Applets is also possible, in particular the popular image processing library ImageJ [20]. However, iLAP does not manage biological data directly, as data remain in files that should be uploaded and associated with a specific experiment or protocol. It is therefore not possible to search for a particular piece of biological data. iLAP does not provide tools for annotating pictures from experimental results such as SDS-PAGE (Sodium Dodecyl Sulfate PolyAcrylamide Gel Electrophoresis) gels or micro-plates, nor does it provides facilities to generate reports.

#### SLIMS

Daley et al. [14] developed SLIMS, a Sample-based Laboratory Information Management System. SLIMS is a web application that provides members of a laboratory with an interface to view, edit, and create sample information. Unlike iLAP, SLIMS leverages the relational database to store and manage biological data. However, its web-interface does not utilize recent advances in web technologies. For example, most data are displayed to the user as static HTML tables, which cannot be dynamically mined nor customized. SLIMS also features a micro-plate annotation tool. Micro-plate pictures, though, cannot be uploaded nor visualized along with their annotations. Similarly, SLIMS supports SDS-PAGE gels, which can be downloaded as plain text files, but may not be properly visualized using the picture of the gel. Reports can be generated and exported, but cannot be customized.

In this paper, we propose the EnzymeTracker, an open-source web-based laboratory information management system for sample tracking, as an efficient and user-friendly alternative that aims at facilitating entry, mining and sharing of samples and experimental biological data. Our system facilitates sample tracking by using QR matrix barcodes and features advanced yet intuitive biological data annotation and visualization tools as well as a flexible and customizable report designer.

## Implementation

### Architecture Overview

Despite their numerous benefits over spreadsheets, database management systems still lack satisfactory user interfaces for data analysis [21] whereas Excel spreadsheets do provide intuitive and well-known graphical interfaces for data analysis and consolidation, provided the issues mentioned above are addressed.

Web-based applications are dynamic and interactive websites that offer a rich user interface comparable to standard desktop programs [22,23]. They can be executed on any connected workstation, without software installation or specific requirements besides a recent web-browser and an active Internet connection to remotely access data. Most importantly, web applications have the major advantage of being always up-to-date wherever they are being accessed, thereby eluding the need for multiples copies of the same document on different workstations, effectively solving synchronization issues between local copies.

**Figure 1:**
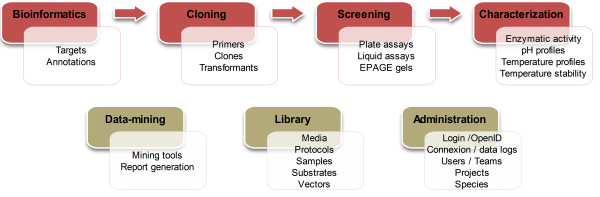
**Overview of the functionalities of the EnzymeTracker**. The EnzymeTracker features a collection of online spreadsheets and tools covering bioinformatics, cloning, screening and characterization. It also provides libraries of shared data such as protocols, and administration tools for data access control using OpenID and user/team management.

The EnzymeTracker was designed to present the acceptance characteristics (see above section ) to maximize its utility. It consists of an integrated and interactive collection of online spreadsheets accessible over the Internet and backed-up by a relational database for efficient data management. Spreadsheets are organized to follow the typical workflow in experimental biology and can be used to track preliminary bioinformatics analyses of potential targets of interests, cloning experiments, screening and expression data (Figure 1). It features a number of novel online tools to facilitate data entry and visualization. The EnzymeTracker also provides a library of shared records such as experimental protocols for sample assays and a comprehensive set of reporting and system administration tools. Unlike commercial LIMS, the EnzymeTracker is open-source. The main benefit for laboratories is that they can extend our system and implement new features to suit their specific needs at no additional cost. With minimal programming skills, it is possible for example to add a simple spreadsheet to accommodate new types of data in a few hours.

We implemented the online spreadsheets as a set of highly dynamic web pages implemented using Asynchronous JavaScript and XML (AJAX) web technologies [23], which enable a web application to communicate with a server in the background using JavaScript and *XMLHttpRequest *objects, without interfering with the current state of the page. AJAX technologies provide an effective means to create dynamic web pages that can interact with the user.

Existing open-source solutions (see section above) were designed using a *fixed *layout. Fixed layouts stay the same width and float on the background regardless of the size of the monitor. It is thus possible for the designer to fine control the parameters of a fixed layout. However, a major downside of fixed pages is that the layout does not accommodate well larger monitors as the layout must accommodate smaller screens as well. For example, at the Full-HD definition (1920*1080), nearly half of the screen is blank when using iLAP, which may be frustrating. To make the EnzymeTracker more accessible, we implemented our system using a *fluid *layout that automatically fits the content to the screen definition of the user. We successfully tested the EnzymeTracker on various screen definitions up to 3840*1080 (dual Full-HD configuration). Our fluid approach will become increasingly beneficial for users as the sizes of monitors have significantly increased in the past few years and large definitions (> 1024*728) now account for over 85% [24].

The implementation of the EnzymeTracker also relies on a number of open-source programming libraries. The web user interface (see section below for an overview) of the EnzymeTracker was implemented using ExtJS v3.0 [25], the general open-source AJAX framework from Sencha. It is backed-up by the freely available MySQL v5.0 relational database management system [26]. The server-side code was implemented using PHP v5.1 [27]. The OpenID authentication relies on the LightOpenID implementation [28]. The visual annotation tools were build using the wz_jsgraphics v3.05 Javascript graphics library [29].

### Reporting

In order to facilitate reporting and data sharing data among collaborators, the EnzymeTracker provides a flexible and user-friendly web interface for designing report templates. A report template is similar to other tables within the EnzymeTracker, except that the user can dynamically select the pieces of information to include in the report. It is also valuable to aggregate data from various tables or consolidate statistical data.

For instance, one can easily create a report template to display the percentage of transformants that were successfully assayed or the molecular weight of a protein.

**Figure 2:**
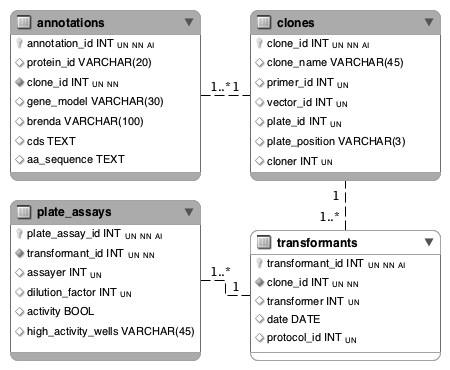
**Simplified Entity-Relationship diagram to illustrate reporting mechanisms**. Foreign keys are marked with solid bullets. Shaded tables are explicitly listed in the configuration of the report. The *transformants *table (white) is not listed but is implicitly required to perform the join query.

Our report designer is particularly useful for more complex queries, such as non-trivial joins when two pieces of information from two tables are not *directly *related and a number of intermediate tables must be used in order to join the two tables. For example, consider the case when the user needs to list the plate assays performed on clones related to cellulase. Figure 2, which illustrates a simplified Entity-Relationship diagram of tables relevant to generate this report, shows that plate assays are performed on transformants, not on clones directly. Fortunately, transformants are related to clones, hence it is possible to define an *implicit *relation between clones and plates assays using transformants. However, manually writing the corresponding SQL query requires a deep understanding of the underlying database structure and more advanced database skills. It is therefore not a viable solution in most biological laboratory.

Instead, we designed Algorithm 1 to automatically compute the optimal implicit joins to relate two tables: when a report is designed, the corresponding SQL query is automatically generated based on the report configuration. In other words, the designer enables scientists with no database expertise to effectively design complex SQL queries using our user-friendly interface and generate fully customized reports that will fit their needs in a few clicks (see section "Report designer" for details about the report designer).

More formally, our algorithm relies on the *findShortestPath() *method derived from Dijkstra's shortest path algorithm [30]. The optimal join is defined as the path between the two tables with the lowest cost. The *cost *of a join between two tables is defined as the length of the shortest path between the two tables in the undirected weighted graph implied by the database structure, where the nodes represent tables and edges, foreign keys. Using the above example, the cost of the join between *clones *and *plate assays *is 2.

**Input**: Explicitly listed tables **tabs**

**Input**: Graph representation of the database **dbGraph**

**Output**: List of tables needed for the join **out**

1 **out, checked **← **array();**

2 **out[0] **← **array (*table ***⇒ **tabs**[0], *from *⇒ *null*);

3 **checked[0] **← **tabs[0];**

4 **for ***i *← 1 **to count(tabs) do**

5     tA ← tabs[*i *-- 1];

6     tB ← tabs[*i*];

7     **if tB ***not in ***out then**

8         path ← findShortestPath(tA,tB);

9     end

10 end

**Algorithm 1**: List of tables needed for the optimal join

Edges were weighted based on the *biological *significance of the foreign keys. For instance, because of the normalization of the database, a number of intermediate joining tables are created to define the relationships between *real *biological entities -- in particular in *m *: *n *relations -- which incorrectly increases the cost of the relationship as the path between the two biologically meaningful tables is longer. The cost of edges in *m *: *n *relations was therefore reduced to avoid the bias induced by the normalization process during the database design.

When the configuration of the report template is updated, it is sent to the server as an XMLHttpRequest object and the optimal join is computed and executed by the SQL engine. The results of the query are finally used to build the configuration of the ExtJS spreadsheet used to dynamically preview the report as the template is being built. Report templates are saved in the underlying database as views.

## Results

### User Interface Overview

**Figure 3:**
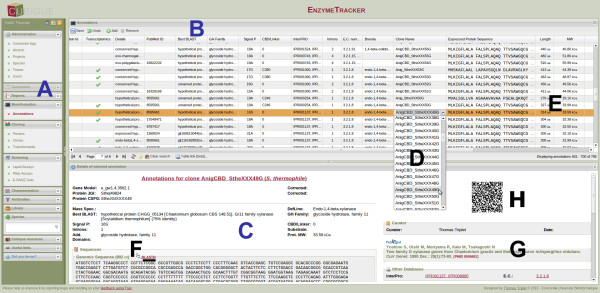
**Overview of the web-based user interface of the EnzymeTracker**. The main menu (A) is on the left. The main panel is usually composed of a spreadsheet (B) and a panel at the bottom to display the entry selected in the spreadsheet using a more readable layout (C). Cross-references to other tables are associated with a combo box, whose content is dynamically generated after the content of the referenced table (D). Cells are automatically computed whenever possible. For example, the length of a protein sequence and its molecular weight (E). References from the literature are automatically fetched given the PubMed ID of an article (G) and jobs for nucleotide or protein sequence alignment can be submitted to NCBI's BLAST server in one click (F). A QR Code that summarizes the record is displayed (H) and may be printed on the sample container for future reference.

Figure 3 gives an overview of the graphical user interface (GUI). Most pages are composed of three panels: the main menu (A) on the left, a spreadsheet (B), which is the primary means to enter to enter data, and a panel at the bottom (C), whose content depends on the data to display. Others data entry means such as those for visual annotations are presented later in this section. Panels A and C can be dynamically collapsed and resized to customize the workspace as needed and the spreadsheets can be displayed in full screen mode to maximize the usable working space thanks to the fluid-layout of our system. Spreadsheets may also be customized by displaying, hiding, reordering and resizing columns as needed so that only the most relevant data are displayed.

The content of the lower panel (C) varies with the data being shown. On most pages, the panel displays the record selected in the spreadsheet in a more readable format. Depending on the spreadsheet, it can provide links to cross-referenced databases such as the Gene Ontology [31] or the Clusters of Orthologous Groups of proteins (COG) database [32]. It also automatically fetches complete references from the literature using PubMed's public API [33] given the PMID of an article (G) and jobs for nucleotide or protein sequence alignment can be submitted to NCBI's BLAST server in one click (F). A QR Code (matrix barcode) also summarizes the current record and may be printed on the sample container for future reference (see more details below).

### Data Entry

The spreadsheet (Figure 3B) is the primary means of entering data in the EnzymeTracker. Each cell is associated with an editor whose format depends on the data within the cell. Most cell editors are simple text fields. More advanced editors are provided where needed. In particular, cross-references to other tables are typically associated with a combo box, whose content is dynamically generated after the content of the referenced table. Figure 3D illustrates the utilization of a combo box to select a clone in the page for *Annotations*. Combo boxes facilitate data entry by suggesting entries as the user types. They also have the added benefit of limiting data entry mistakes, in particular when users enter data that do not exist in the referenced table. Specific editors are also available for Boolean flags and dates. The EnzymeTracker also supports rich text editors with text formatting capabilities, which are mainly used for comments and free-text cells. End-users also have the possibility to undo modifications made in the active spreadsheet using the GUI before saving data, so that typos can be quickly corrected without triggering the versioning and backup mechanism.

#### Data integrity and validation

To further reduce entry errors, each cell editor can be associated with a *validator*. Validators check the correctness of data types and send immediate feedback to the user in case of an error. They are usually based on regular expressions or more advanced customized functions as needed. Validators are also useful to enforce data entry conventions and consistency within a group of users.

In addition, to minimize data entry, cells are automatically computed whenever possible. For example, the length of a protein sequence and its molecular weight (E) are automatically calculated when one enters a protein sequence. Calculated fields are also used to reduce data redundancy compared to standard spreadsheets. For instance, the name of a protein should appear on several related spreadsheets. Using standard spreadsheets, the user needs to copy/paste the name of the protein wherever needed. This will lead to inconsistencies between spreadsheets during their update. In the EnzymeTracker, the underlying relational database is leveraged to display the name of the protein in all tables where it is needed. The first benefit is that the protein name is automatically displayed whenever there exists a relation between proteins and the current spreadsheet. Second, changes to the protein data are automatically reflected in all tables. Data in the various online tables are therefore always consistent and up-to-date.

#### Data importation/exportation

In some cases, the different enzyme assays and characterization of samples were already being recorded using Excel spreadsheets. We therefore implemented importation routines to facilitate the migration process to the EnzymeTracker. From experience, basic data importation by uploading and parsing files is error-prone, as files formats and layouts tend to vary between files. For example, one column may be missing in one file, which will shift other columns and lead the parser to import the wrong data.

Instead, we implemented a drag-and-drop importation mechanism where appropriate. The user selects the data to import in the Excel file and drags and drops the selection into the browser's window. The major benefit of this semi-automatic approach is that it makes it easier for the users to review the data before importation, hence reducing the number of errors made. It also gives more flexibility as only specific records can be selected and imported. Finally, users have the possibility to export EnzymeTracker spreadsheets to Excel documents in one click. Data may also be imported programmatically, using JavaScript and RESTful requests.

### Versioning and backups

Our system is supported by a relational database, which efficiently handles versioning and backups. Unlike in standard spreadsheets, when a user updates or deletes a record in the EnzymeTracker, existing data are always backed-up and flagged as obsolete so that is it not displayed in the web GUI. The modifications are also logged for future reference as part of the record's history, which is displayed in the lower panel of the interface (Figure 3C). The history of the record is also accessible when generating customized reports (see Section *Reporting*). As a consequence, while updating a spreadsheet is always possible, no data are ever deleted and restoring a record to a previous state or accessing the complete data modification log in case an error is made while updating a spreadsheet is always possible.

### Visualization tools

Most data in the EnzymeTracker can be viewed using tables. In a number of cases however, tables may be improved to give the user a more visual perspective of the data. To enhance the utility of experimental screening data, the EnzymeTracker integrates a number of annotation and visualization tools. The following sections describe how the bottom panel of a spreadsheet (Figure 3C) can be customized to accommodate plate assays and E-PAGE^TM^48 gels from Invitrogen respectively. While the EnzymeTracker does not natively support other techniques, new functionalities and new tools that suit the specificities of each laboratory can be easily implemented with minimal web development skills.

#### Micro-plate assays

Micro-plate assays are widely used in molecular biology and high-throughput screening to simultaneously test multiple samples for their responses to chemicals, living organisms or antibodies or to detect the presence of particular proteins or gene sequences. Those responses are typically quantified by measuring the fluorescence or color changes in markers associated with compounds on the plates. The plate assay is usually repeated twice, at two different sample dilution factors.

**Figure 4:**
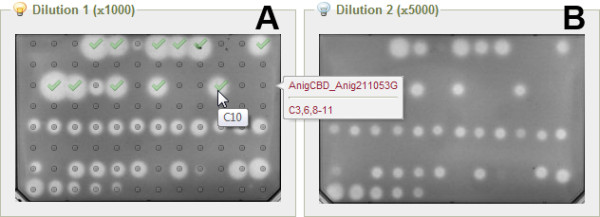
**Graphical user interface for the annotations of plate assays**. Pictures of the micro-plates for the two dilutions can be uploaded and automatically annotated based on the content from the tables describing *clones *and *transformants*. High-activity wells can be selected within the web interface by clicking on picture. Annotations can be laid over the picture (A) or hidden (B) as needed.

The EnzymeTracker enables users to upload the two micro-plate pictures for the two dilutions of each experiment (Figure 4). The tables describing *clones *and *transformants *are leveraged to automatically annotate the plate. A "virtual plate" representing the 96 wells can also be layered over the original picture (A) or hidden (B) as needed. The virtual plate is also convenient to quickly visualize and identify most active wells by simply clicking on the desired wells directly on the picture.

#### SDS-PAGE gels

**Figure 5:**
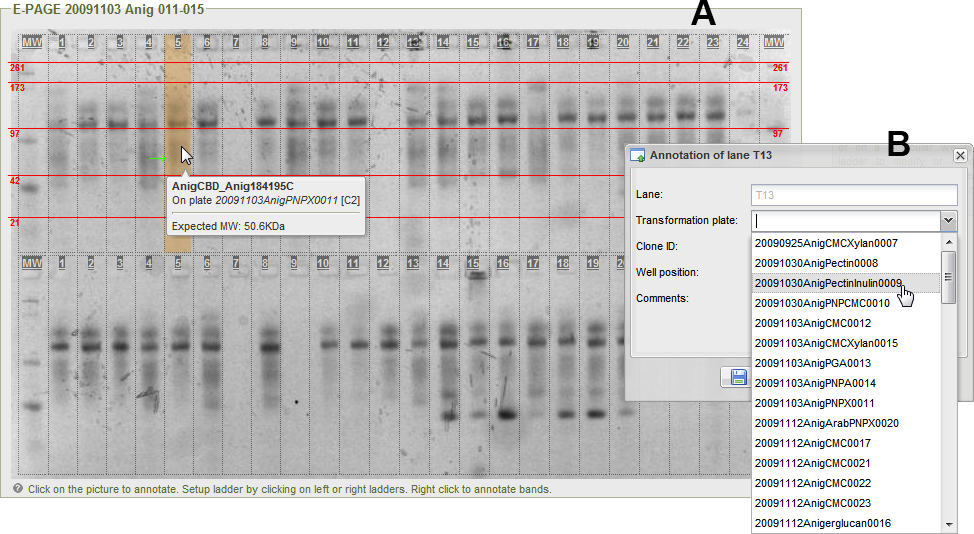
**Graphical user interface for the annotations of E-PAGE™48 gels from Invitrogen**. Pictures of the gels can be uploaded and annotated within the web interface of the EnzymeTracker.

E-PAGE^TM^48 gels are improved SDS-PAGE (Sodium Dodecyl Sulfate PolyAcrylamide Gel Electrophoresis) gels broadly used for high-throughput protein separation and analysis. Each gel comprises 48 lanes for samples and 4 marker lanes, which define the ladder of the molecular weights of the proteins on the gel. Similarly to plate assays, the picture of the gel can be uploaded and annotated within the user interface (Figure 5). Each sample lane in the gel (A) can be annotated using a form (B) that is displayed upon click. A tool-tip summarizing annotations of a lane is displayed when hovered by the cursor (orange). The dropdown menus in (B) to select the clone and the transformation plate loaded in each lane are dynamically built based on their respective tables. In addition, specific bands can be highlighted (green arrows) and annotated. Finally, the ladder (red) can be easily setup by clicking on one of the four outer marker lanes.

The EnzymeTracker thus reduces the need for external tools and leverages data from other spreadsheet to facilitate the annotations of hundreds of experimental data and to reduce data entry errors.

#### Chart visualization

In many cases, the experiments aim at characterizing the evolution of a variable given a set of parameters. Representing the data using charts is then is suitable alternative to tables for data presentation.

**Figure 6:**
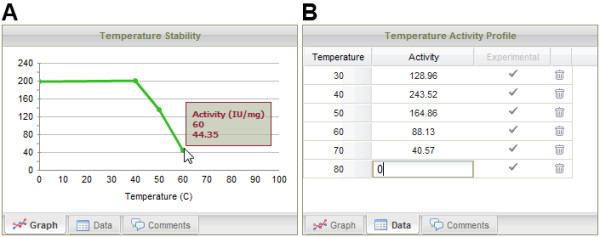
**Visualization of experimental data using charts**. The EnzymeTracker can use charts to visually represent characterization data. Each chart panel includes a tab for the graph (A), the underlying data in a table (B) and free text to comment on the chart (not shown). Data may be imported in the table (B) from Excel or edited within the interface.

Figure 6 illustrates the use of charts in the EnzymeTracker, using the example of the characterization of the activity of a sample as a function of its temperature. Each chart panel is a composite of three sub-panels: the graph itself (A), the underlying data in an editable table (B) and a free-text field for comments and additional information regarding the chart (not shown). Graphs are usually represented using curves although histograms and pie charts are also supported. The graph is dynamically updated when the underlying data is edited within the interface or imported from Excel as described above.

Graphs are also used in the administration console, in particular to display connection and data logs.

### Sample tracking with QR Code

For each record, a Quick Response (QR) Code is generated using Google Chart API [34] and displayed on screen. QR Code is a type of matrix barcode that can be used to encode up to 4,296 alphanumeric characters. Its specifications were disclosed and it was published as an ISO standard (ISO/IEC 18004:2006). The EnzymeTracker uses QR Codes to summarize the content of the record. The pictogram can be downloaded at a higher resolution and printed on the sample container for future reference. It can then be read as needed by inexpensive scanners or any reasonably recent smartphone, useful in laboratories, as space on the bench is often limited.

### Data-Mining and Reporting

As of June 2011, over 55,000 entries have been saved within the EnzymeTracker and a growing number are being recorded on a daily basis. While the EnzymeTracker should not be considered as a complete framework for data warehousing and data integration of complex biological types, it features user-friendly tools that enable scientists to easily mine for specific pieces of information among large amounts of heterogeneous data. For example, a principal investigator may look for "all enzymatic activities detected during liquid assays performed by his assistants in the past two months on clones from *S. thermophile*".

#### Context-dependent filtering

**Figure 7:**
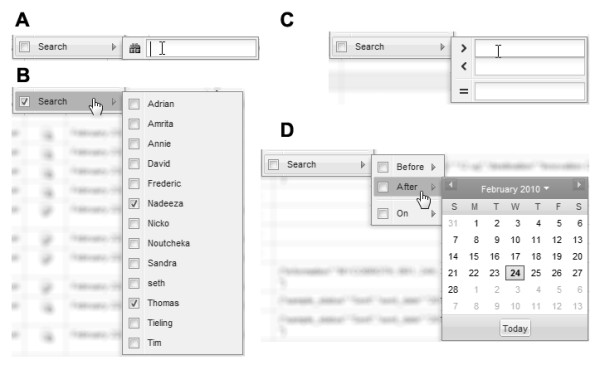
**Data mining**. Spreadsheets within the EnzymeTracker are fully searchable. Each column is associated with a filter that depends on the type of data the column represents. Five kinds of filters are available: textual (A), multi-selection (B), numerical (C), calendar (D) and Boolean (not shown).

Each table in the EnzymeTracker is fully searchable and each column is associated with a flexible filter that depends on the type of data the column represents. Five different types of filters can be configured as shown on Figure 7: textual (A), multi-selection (B), numerical (C), calendar (D) and Boolean (not shown). Numerical filters let the user query for values above, below or equal to a given threshold. They are most useful to query biochemical properties of enzymes and samples, for example protein sequence length or molecular weight, or the temperature stability of a molecule. Boolean filters are typically used to retrieve records when given a flag. For instance, this filter is convenient to list all assays where a strong activity has been reported. Calendar filters are helpful to search for records given a time frame. The multi-selection filter is most effective for searching for one or more items in a given list. The list may be static or may be dynamically generated by the server based on data from other tables. For example, it is possible to search for samples from a given organism, the list of organisms being automatically generated by the database server.

#### Report designer

**Figure 8:**
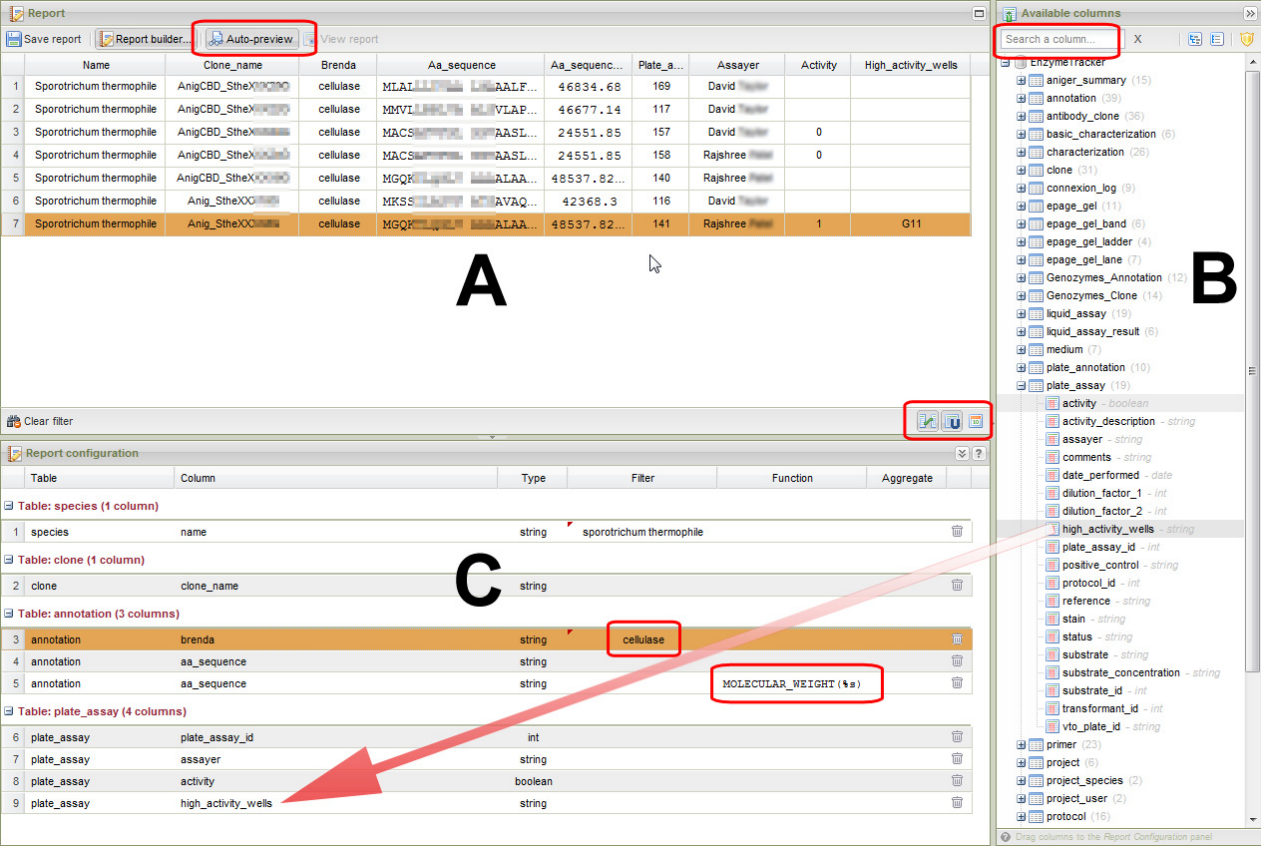
**User interface for the report designer**. The central panel (A) displays a preview of the report. The right-most panel (B) shows a searchable list of items that can be dragged and dropped in the configuration panel (C). The configuration of the report template can be further refined by filtering data, applying various functions or aggregating data. A number of flags to fine tune SQL queries are available for advanced users. A preview of the report can be automatically generated when the configuration is updated to facilitate the creation of the report. Once created, reports are automatically updated and may be further mined.

The web-based user interface for designing report templates is shown in Figure 8 and comprises three main panels. The right panel (B) lists all items that can be included in a report. Items are grouped based on the spreadsheet where they can originally be found. The list is searchable so that relevant pieces of information can quickly be assembled together. To create a report, one needs to drag-and-drop the desired items from the list to the lower configuration panel (C). A preview of the report can be automatically displayed in the central panel (A) when the configuration of the report changes. The report can also be refined using flags, for example to decide whether to display only current values of a record or its modification log also.

Once a template is created, it can be shared and displayed like other tables. In particular, the report can be further refined using filters as described in the previous section. In addition, reports are automatically updated as more data is added to the EnzymeTracker: there is therefore no need to re-design a report to display up-to-date data. Finally, reports can be easily shared with collaborators or saved as standard Excel files for further analysis.

## Conclusions

The EnzymeTracker was designed to be flexible, easy to use and offers many benefits over spreadsheets, thus presenting the characteristics required to facilitate acceptance by the scientific community. Unlike expensive commercial software, the open-source license of the EnzymeTracker allows laboratories to easily extend the EnzymeTracker as needed to fit their specific needs. Our system has been successfully used for 20 months on a daily basis by over 50 scientists to monitor protocols and experiments conducted to identify, annotate and fully characterize thousands of samples from 20+ fungal species.

The initial implementation of the EnzymeTracker has focused on facilitating sample tracking and experimental data annotation and visualization. The future development of the EnzymeTracker will focus on the implementation of widgets based on the online spreadsheets, which will facilitate data sharing as widgets can be embedded in virtually any web page. Widgets will also facilitate the development and sharing of new functionalities to support additional data types and material or protocols by the community. We will also enhance reporting by allowing chart generation in addition to tabular data.

## Availability and requirements

The EnzymeTracker and its documentation are available at http://cubique.fungalgenomics.ca/enzymedb/index.html under the GNU General Public License v3.

• **Project name**: EnzymeTracker

• **Project home page**: http://cubique.fungalgenomics.ca/enzymedb/index.html

• **Operating systems**: Platform independent

• **Server requirements**: Apache 2, MySQL 5.0, PHP 5.1.6

• **Web browser requirements**: Chrome, Firefox 3.5+, Safari 4+

• **Programming languages**: HTML, Javascript, PHP

• **License**: GNU GPL3

• **Any restrictions to use by non-academics**: None

## List of abbreviations

AJAX: Asynchronous JavaScript and XML; API: Application Programming Interface; BLAST: Basic Local Alignment Search Tool; GNU: "GNU" is Not Unix (recursive acronym); GPL: General Public License; GUI: Graphical User Interface; ISO: International Organization for Standards; LIMS: Laboratory Information Management System; QR Code: Quick Response Code; REST: REpresentational State Transfer; SDS-PAGE: Sodium Dodecyl Sulfate PolyAcrylamide Gel Electrophoresis; SQL: Structured Query Language; XML: Extensible Markup Language;

## Authors' contributions

TT designed and implemented the system. GB defined the requirements of the system and devised the backup and versioning mechanisms. TT and GB drafted and approved the final manuscript.

## Endnotes

a: Licence fees were obtained on GSA on September 19, 2011 at: http://www.gsaadvantage.gov/. These prices represent the lowest possible prices negotiated with the U.S. government and may not be available to non-governmental companies or organizations.

## Supplementary Material

Additional file 1 **Brief review of 15 open-source LIMS referenced by goomedic.com**. Practical and free LIMS are extremely limited. We briefly reviewed a few open-source projects referenced by goomedic.com. First, it should be noted that open-source projects are not necessarily free to use: 2 of the systems were not 100% free for the end-user. More than half of the projects (53%) are not practical solutions because they are still in early development stages or not stable enough to run without crashing (including 3 projects which are not supported any more). 6 projects were simple clinical trials or inventory/order management systems and were not designed to track experimental biological results. One system was even designed to reduce travel expenses related paperwork. While lightweight and functional, ms lims was designed for the tracking and analysis mass spectrometry data only. Click here for file
