# Supplementary material for: The EnzymeTracker: an open-source laboratory information management system for sample tracking
Source: BMC Bioinformatics. 2012 Jan 26;13:15. doi: 10.1186/1471-2105-13-15 (PMC3353834; doi:10.1186/1471-2105-13-15)
Supplement: Additional file 1 — Brief review of 15 open-source LIMS referenced by goomedic.com. Practical and free LIMS are extremely limited. We briefly reviewed a few open-source projects referenced by goomedic.com. First, it should be noted that open-source projects are not necessarily free to use: 2 of the systems were not 100% free for the end-user. More than half of the projects (53%) are not practical solutions because they are still in early development stages or not stable enough to run without crashing (including 3 projects which are not supported any more). 6 projects were simple clinical trials or inventory/order management systems and were not designed to track experimental biological results. One system was even designed to reduce travel expenses related paperwork. While lightweight and functional, ms lims was designed for the tracking and analysis mass spectrometry data only. [file 1471-2105-13-15-S1.DOC]

**Annex 1:** Review of the 15 LIMS referenced by goomedic.com

| **Status** **LIMS** (alphabetical order) | **Dead** | **Early development** | **Buggy/ Crashing** | **Not tracking *experimental biological* data** | **Specific to one type of data only** | **Not 100% free** | **Comments** |
| --- | --- | --- | --- | --- | --- | --- | --- |
| **BikaLIMS2** |  |  |  | **X** |  | **X** | - *Bika LIMS* has thus far been perceived mainly as leading open source chemistry LIMS with applications in agriculture, food and beverages, environmental monitoring and water quality management. - Basic functions are free. Optional modules and support at a cost. - Requires the *Plone* content management system, which “is not a trivial software stack” and runs best on dedicated server (hidden cost). |
| **Electronic Lab Notebook** |  |  | **X** |  |  |  | - Doesn’t start: *ELN* is not able to open shared library libc.so.6. - Server is listening to port 1234, well known to be unsecure, as malware programs have used it to infect systems. |
| **eyeLIMS** | **X** |  |  |  |  |  | - Last update: 2009/07/17. - Code not available for download. |
| **FlowCytometryLIMS** |  |  | **X** |  | **X** |  | - Unable to install. - Limited to flow cytometry experiments. |
| **FreeLIMS** |  | **X** | **X** |  |  | **X** | - Current version crashes when logging-in (log reports missing libraries) - Next release will *not* be free. |
| **HalX** | **X** | **X** | **X** |  |  |  | - Last update: 2009-09-29. - Very early development stage. |
| **LabLog** |  | **X** | **X** |  |  |  | - Still in early development. - Too buggy for thorough testing. |
| **LabStoRe** |  |  |  | **X** |  |  | - Inventory management system. |
| **ms_lims** |  |  |  |  | **X** |  | - Lightweight and functional. - Designed for – and only for – mass spectrometry data. |
| **OMIL** |  |  |  | **X** |  |  | - Travel expenses management system. |
| **OpenELIS Global** |  |  |  | **X** |  |  | - *OpenELIS* is dead. Considering the fork *OpenELIS Global* for review - Designed to track inventory and clinical tests (HIV) on patients. |
| **Open-LIMS** |  | **X** |  |  |  |  | - The available version is “not recommended to use it in any kind of productive environment”. |
| **protLIMS** | **X** |  | **X** |  |  |  | - Last update: 2006-05-19. - Unable to run it. |
| **Science Lab Inventory** |  |  |  | **X** |  |  | - Inventory and order management system. |
| **YouLabData** |  |  |  | **X** |  |  | - Inventory and order management system. - Data stored remotely on author’s server, with no guarantee regarding the safety of the data (the author even recommends saving the data elsewhere). |
|  | **20%** | **27%** | **40%** | **40%** | **13%** | **13%** | Note: Percentage of projects for each status. |
